# Tau‐targeting therapeutics: the next frontier in disease‐modifying treatments for Alzheimer’s disease

**DOI:** 10.1002/alz70859_099833

**Published:** 2025-12-25

**Authors:** Szofia Bullain

**Affiliations:** ^1^ Biogen, Cambridge, MA USA

## Abstract

The clinical development of tau‐targeting therapies for the treatment of Alzheimer’s disease (AD) represents a promising new frontier for the field. While the pathophysiological role of tau in AD is incompletely understood, multiple intervention strategies targeting aggregated or extracellular forms of tau protein or tau expression are being explored. To date, most tau‐targeting therapies intervene at three core elements of tau physiology and pathophysiology: production, aggregation, and propagation. Current therapeutic approaches to targeting these elements include vaccines and monoclonal antibodies, oligonucleotide‐based therapeutics, gene therapy, and small molecules. So far, results from these strategies have yielded varied outcomes, highlighting both challenges and opportunities in the clinical development pipeline. Despite the failure of several anti‐tau monoclonal antibodies to demonstrate clinical efficacy, the field continues to evaluate antibodies that target different tau epitopes. It has been hypothesized that targeting the mid‐region of tau might interfere with the cell‐to‐cell propagation of pathogenic forms of tau; however, it is unknown if slowing extracellular tau accumulation will result in improved clinical outcomes. Additionally, learnings from a clinical trial that evaluated an antisense oligonucleotide targeting microtubule‐associated protein tau (MAPT) messenger RNA, which encodes tau, suggest that decreasing the production of tau protein might provide clinical benefit. This presentation explores key lessons from recent clinical successes and setbacks, offering critical insights to guide the future development and integration of tau‐targeting therapies into the AD treatment paradigm.

